# Cavitation activity induced by spring-loaded core needle biopsy devices

**DOI:** 10.1038/s41598-025-97497-z

**Published:** 2025-05-06

**Authors:** Jussi Kiviluoto, Maxime Fauconnier, Heikki J. Nieminen

**Affiliations:** Medical Ultrasonics Laboratory (MEDUSA), Department of Neuroscience and Biomedical Engineering, Aalto University, 02150 Espoo, Finland

**Keywords:** Needle biopsy, Spring-loaded core needle, Cavitation, Diagnosis, Applied physics, Fluid dynamics

## Abstract

Core needle biopsy is a common medical procedure to obtain tissue samples with tissue architecture for pathological assessment. One prevalent method involves the use of spring-loaded core needle biopsy devices, or “biopsy guns”. Despite their intense motion dynamics when shot through tissue, possible cavitation activity has received limited attention. Cavitation bubbles imploding in biological environments are known for their mechanical effects on cells and tissue. In this study, visual and acoustic monitoring was applied to characterize and quantify cavitation phenomena around longitudinally or flexurally oscillating core needle biopsy needles, when immersed in deionized water or embedded in agarose-based tissue mimicking phantom. In water, we observed that cavitation was most prominent with side cut needle, but bubble activity was also present with front cut needle. In agarose, the intensity of the cavitation was found to decrease with increasing agarose concentration. Cavitation was still observed at 0.3% w/v agarose gel, but at 1.0% w/v gel, cavitation activity was essentially eliminated. Acoustic emission was observed with both needle types from audible to ultrasound ranges. The study suggests that cavitation as a physical mechanism can occur in operation of spring-loaded core needle biopsy devices in water and tissue-mimicking hydrogels and should be considered as an opportunity for the development of new in vivo applications related to the echogenicity of the cavitation bubbles in ultrasound imaging as well as considered as a physical mechanism for safety studies.

## Introduction

Core Needle Biopsy (CNB) is a medical procedure for obtaining a tissue sample directly from a target tissue for pathological examination^1^. This is achieved by the use of coaxial needles including a reservoir designed to capture a tissue construct cut out from the target tissue^1^. CNB is largely considered as a time efficient, relatively safe and affordable procedure that provides healthcare professional with accurate diagnostic information through pathological assessment^2^. This in combination with the feasibility of CNB at various anatomical sites (e.g., breast^3^, liver^4^, lung^5^ and prostate^6^ tumors) makes them a common and well-accepted procedure widely conducted in hospitals.

CNB can however be limited by the amount of tissue that can be sampled at once due to the fixed size of the sample reservoir. Inadequate amounts of sample tissue, unsatisfactory sample quality, or misguided operation of the devices can result in the need of more sampling instances^7^. This could lead to increased patient discomfort and greater risks for complications such as hemorrhage. Other needle biopsy types exist, such as the common fine-needle aspiration biopsy (FNAB)^1,8^, but it can be associated with inadequate yield in up to 1/3 of cases^1^. The more recent ultrasound-enhanced fine-needle aspiration biopsy (USeFNAB) facilitated by the flexural standing waves of the needle could alleviate the issue of inadequate tissue yield by providing energy to remove cells and tissue constructs from the target site^9^.

Medical needles actuated at ultrasonic frequencies can cause cavitation bubbles to emerge in liquids and soft tissues even at low total acoustic power, as recently demonstrated in bovine liver by Perra et al.^10^. Moderate cavitation can be beneficial in fine-needle biopsy, because it can increase tissue yield without compromising sample quality^10^. However, if violent, the stochastic behaviour of cavitation bubbles is widely reported to be able to induce tissue damage^11^. As a result, cavitation, if present, could have implications to safety and performance of needle biopsy devices. Prevalent type of CNB instruments are spring-loaded devices, also referred to as “biopsy guns” or “automated biopsy devices”, which according to the authors’ knowledge have not been associated with cavitation activity in the previous literature. These spring-loaded CNB devices work by first allowing the user to store potential energy in a spring, before releasing it as kinetic energy by accelerating a hollow medical needle structure rapidly into the target tissue. As it reaches its ultimate position, the fast-moving needle structure then comes to an abrupt stop with the help of a stopping mechanism. This further results in significant positive and negative accelerations along both the longitudinal and lateral axis of the needle^12^. Thereby, the intense motion dynamics of CNB devices cannot be excluded as a source of cavitation phenomena.

Generally in hydrodynamics, cavitation bubbles have been associated to the pressure drop located downstream of a mechanical obstacle placed in a fluid flow^13^, which is a configuration similar to the one of a needle moving laterally in a fluid at rest. The potential for induced cavitation, when the liquid is moving with respect to the obstacle, can be estimated by the dimensionless cavitation number *Ca* defined as

$$\begin{aligned} Ca = \frac{p-p_v}{\frac{1}{2}\rho u^2}, \end{aligned}$$

where $\rho$ and $p_v$ are the density and vapor pressure of the liquid, *u* is the flow speed and *p* is the local pressure. Low *Ca* values indicate a greater probability of cavitation. For instance, Wu et al.^14^ observed cavitation inception in the wake of a flow around a wedge-shaped bluff body at $Ca = 4.8$. While the presence of cavitation bubbles can affect the flow, *e.g.* via microstreaming^15^ and jetting^16^, the fluid viscosity, obstacle geometry and flow speed are also relevant parameters^13^. Classically, fluid flow behaviour around an obstacle can be characterized using the dimensionless Reynolds *Re* number,

$$\begin{aligned} Re = u L \rho / \mu \end{aligned}$$

where *L* is a characteristic dimension of the obstacle and $\mu$ is the dynamic viscosity of the fluid ($\mu = 1$ mPa$\cdot$s for water at $20^{\circ }$C). In addition to these two variables, the fluid flow in the wake of an obstacle depends on the geometry of the said obstacle. A hydrodynamic shape facilitating smooth flow around the obstacle will promote a laminar wake downstream, whereas an uneven and edged shape can lead to a turbulent regime in the wake of the obstacle. In that regard, Asano et al.^13^ have shown that a phase transition from a laminar to a turbulent flow ($49 \le Re \lesssim 190$), resulting in the formation of Karman vortices, can take place away from the obstacle at a distance that varies with fluid temperature, viscosity and the possibly induced cavitation bubbles. The complete transition to the turbulent regime occurs in high Reynolds number situations, $Re > 190$^13^.

This study aims to investigate the action dynamics of spring-loaded CNB devices and their induced fluidic effects as a possible source of cavitation activity in water and tissue-mimicking material, which to the authors’ knowledge is a phenomenon not systematically studied or documented. This work presents concurrent video and audio signals to quantitatively compare the results of the action of the spring-loaded devices, when operated inside deionized water. An additional investigation is also performed in agarose gels, used as tissue phantoms representing biological tissue as a site facilitating cavitation inside the human body. The focus is on the evaluation of the impact of viscosity on the intensity and prevalence of cavitation. Eventually, we quantify the sound emission from the system in audible and ultrasound ranges.

## Materials and methods

### Investigated devices

**Fig. 1:**
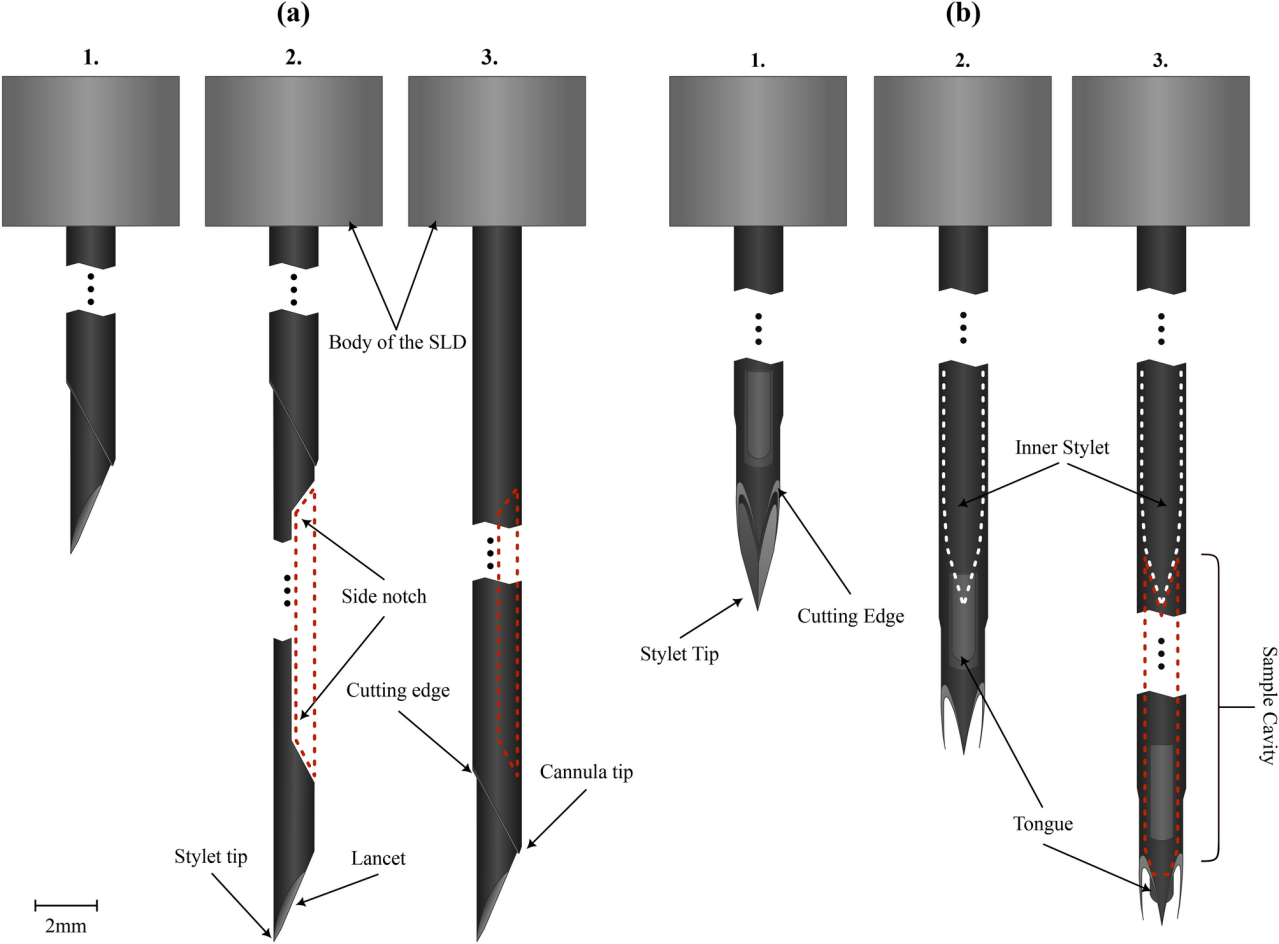
Schematics of the two types of spring-loaded core needle devices (SLDs) employed in this work, i.e., a side cut model (**a**) and a front cut model (**b**) 14 gauge spring-loaded core needle biopsy device, with their principles of action.

The set of spring-loaded CNB devices used in this work can be categorized into two common categories, side cut and front cut style needles, whose dynamics of operation differ and are schematised in Fig. 1a, b, respectively. Simpler in design, the side cut models employ a sharp lancet shaped inner stylet fitted with a non-symmetric notch that deploys before a beveled outer cutting cannula slides across it. This allows the capture of a tissue sample inside the notch in the inner stylet, as the outer cannula seals the notch shut cutting the sample from the surrounding tissue in the process (Fig. 1). The more sophisticated front cut models only have one major moving part, the cutting cannula fitted with a so-called Franseen style needle tip. The cannula structure includes a coaxial pincer, in essence a small and round blade, to separate the sample from the surrounding tissue at the location of the cannula tip after its full deployment. The inner stylet does not move during the operation in the front cut models, its purpose is simply to ease the insertion of the device to the desired location.

We explored a range of spring-loaded devices, with side cut or front cut needle types available in the country of the study. However, no fundamental difference between models within their respective type, side cut or front cut, were identified. Therefore, this study was limited to investigating a 14 gauge, abbreviated as “G”, (14 G = 2.109 mm outer diameter) side cut and 14 G front cut model example.

### Experimental arrangement

**Fig. 2:**
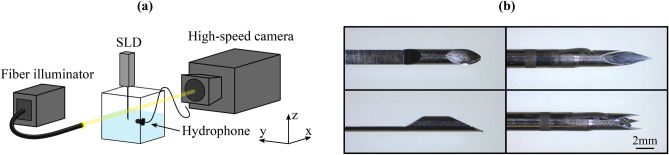
(**a**) Schematics of the experimental arrangement. A spring-loaded CNB device is held in place, so that its needle, and a hydrophone pointed at it are submerged inside deionized water. A high speed camera is focused on the needle tip, while a fiber illuminator is providing sufficient lighting facilitating shadowgraphy. (**b**) Microscopy images of the geometries of the needle tips. On the left, the inner stylet tip of a side cut model, and on the right, the tip of a front cut model in the loaded (above) and deployed state (below).

The experimental arrangement (Fig. 2) consists of a transparent container filled with deionized water (18.2 M$\Omega$ cm). A spring-loaded CNB device was held in place and positioned so that its tip was submerged even in the loaded state (before firing). In addition, doing so would limit the potential formation of cavitation bubbles to originate from the needle contacting the water interface and confirm that all notches and cavities of the needle structure are also largely pre-filled with the DI water before deployment. A high-speed camera (model: Phantom v1612, Vision Research Inc., Wayne, NJ) was placed outside the water container pointing toward the location of the final stop of the needle tip. A broadband hydrophone (model: AS-1, 4Hz-100kHz, Aquarian Audio & Scientific, Anacortes, WA), was submerged inside the water container, pointing towards the final stop of the needle at a distance of 10 cm along the lateral axis. This allowed us to respectively capture the dynamics of the needle and the possible cavitation bubbles (sampling rate 100 kHz, 0.357 $\upmu$s exposure time, 384 $\times$ 288 resolution, scaling 1 pixel = 25 $\upmu$m) and their acoustic emission. A fiber illuminator (model: OSL2, Thorlabs, Newton, NJ) was used to provide sufficient lighting for the camera. The raw hydrophone signal was recorded by the means of a digital oscilloscope (model: InfiniiVision DSOX3014T, Keysight Technologies, Santa Rosa, CA), which was used with a pre-set threshold as a trigger for recording with the high-speed camera, allowing concurrent and co-registered audio and video signal capture. In addition, the moment the oscilloscope triggered was set as the 0 s point on the time axis, which temporally corresponds to the moment when the needle reached the far end position after firing.

In order to assess the possible formation of spring-loaded CNB device induced cavitation bubbles in tissue-like material, similar experiments were conducted within agarose gel (0.3 & 1.0 % weight/volume, Fisher Bioreagents) prepared into phosphate buffered saline at a temperature of $70^\circ$C. The agarose gel concentrations of 0.3 % and 1.0 % w/v were chosen for their similar mechanical properties as soft tissue^17,18^, allowing the gels to mimic soft tissue as a site facilitating cavitation activity. However, the gel prepared considering these mechanical properties, compression modulus and water content, does not allow for tissue damage assessment. The experimental arrangement for these agarose gel trials compromised the use of a hydrophone, and the cavitation dynamics were only evaluated optically using the high speed camera.

### Signal analysis

All recorded audio signals and video were analyzed using Matlab (R2021b). The audio signal was analyzed in the frequency domain by constructing a power spectral density spectrogram, converted to a sound pressure level spectrogram with 1 µPa as the reference pressure, using a short-time Fourier transform algorithm^19^. A window size of 2560 data points with 2500 data points of overlap was used. This allowed us to visualize and investigate all emerging frequency components found in the acoustic emission as a function of time up to 100 kHz.

The video was analyzed to extract the lateral and longitudinal displacement of the needle, as well as the projected two dimensional area of cavitation bubbles. The information of the needle displacement was obtained using a minimum eigenfeatures algorithm^20^, followed by a Kanade-Lucas-Tomasi feature tracking algorithm^21,22^ for the detection and tracking of the important features of the needles, namely the tips of the outer cannula and inner stylet for the side cut models, and the tip of the cutting cannula for the front cut models. The use of these algorithms was facilitated by their implementation in Matlab’s Computer Vision Toolbox (R2021b). The corresponding velocity and acceleration vectors were obtained by computing the derivative with respect to time of the displacement data, once for velocity, and twice for acceleration.

**Fig. 3:**
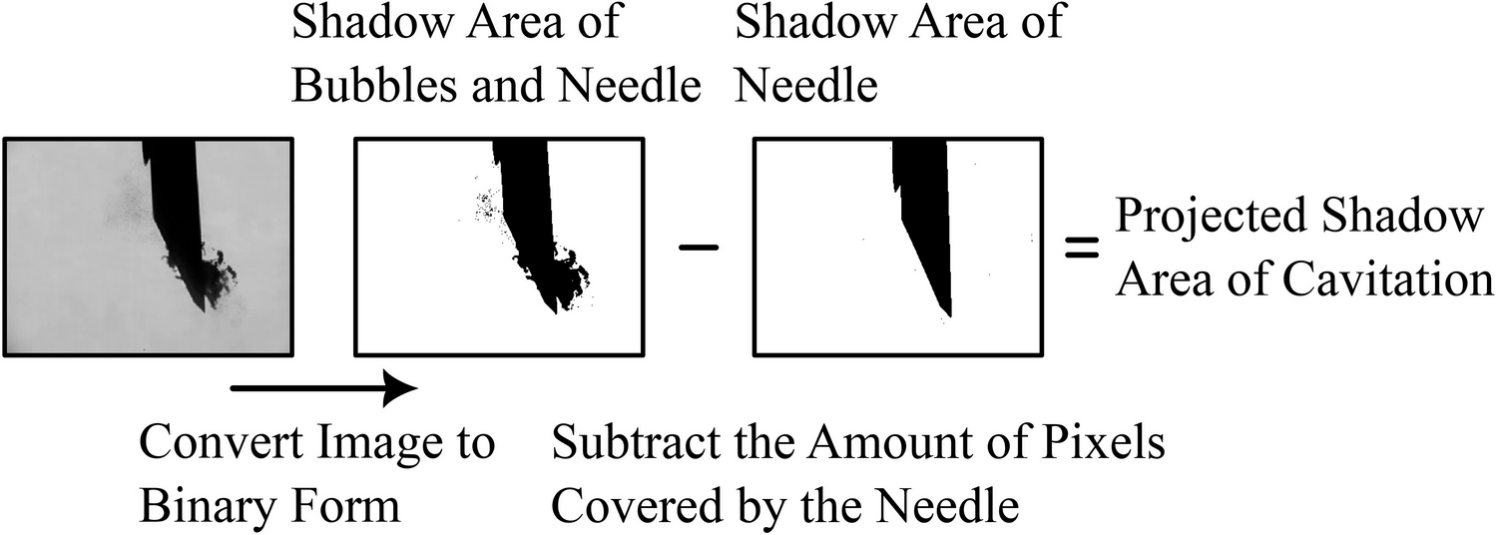
Example of the typical steps of image analysis for approximating the amount of cavitation bubbles. The original images are first converted to binary images, then subtracted by the area covered at rest by the needle only.

Due to the experimental arrangement, the quantification of cavitation activity is limited to an orthogonal projection of the visible bubbles on the *yz* plane. The analysis method is as follows. As illustrated in Fig. 3, the initial step consists in converting each image of the video sequence into a binary form. The area covered by bubbles is then estimated by subtracting the number of pixels covered by the needle at rest from the total number of dark pixels in each binary image.

## Results and discussion

### Side cut needle model

**Fig. 4:**
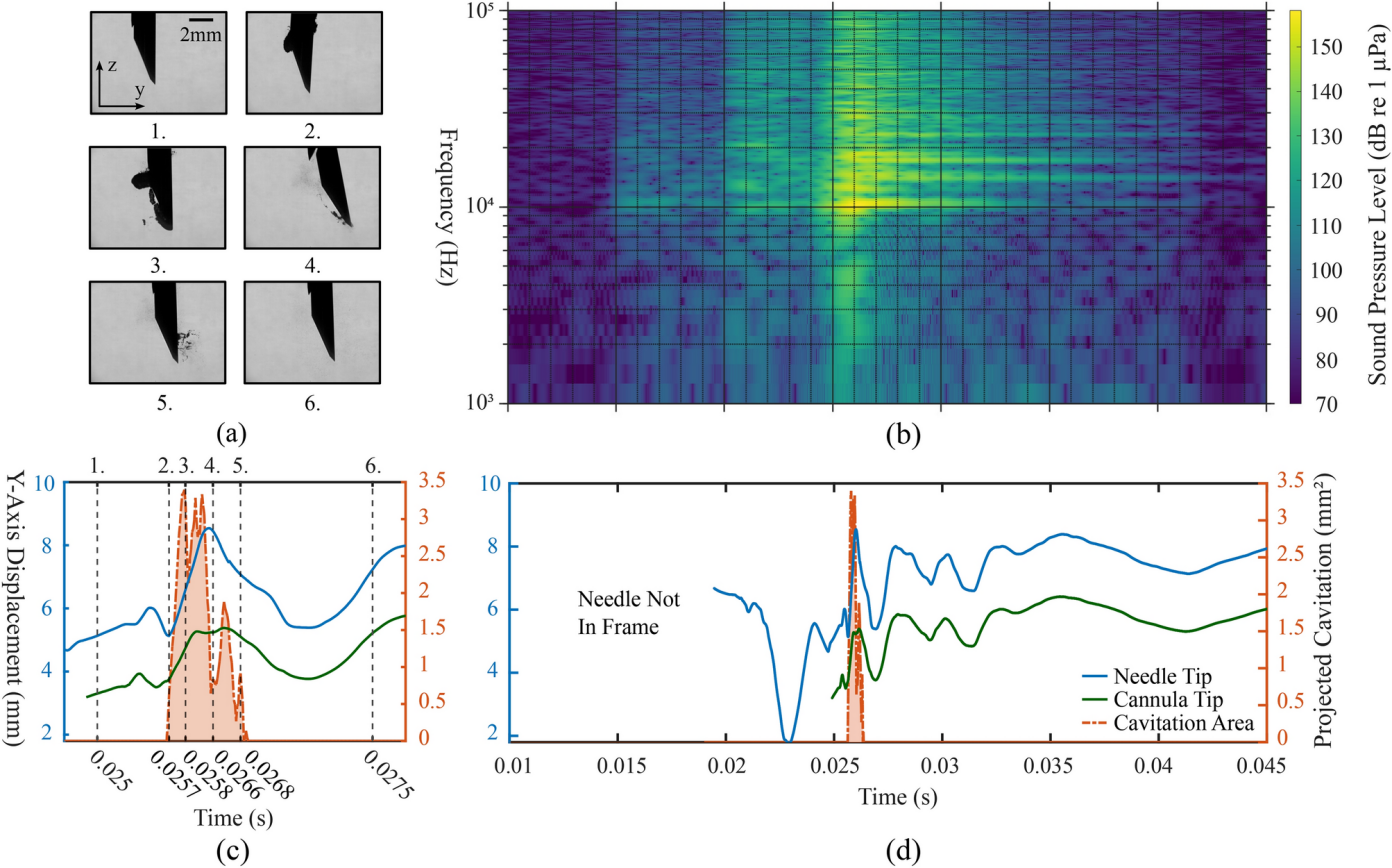
Results of a 14 gauge side cut model needle shot inside deionized water. (**a**) Exemplary images from the high speed video (Supplementary Video 1, total frame size 9.6 mm $\times$ 7.2 mm). (**b**) Spectrogram of the acoustic emission. (**c**) Time interval of interest from (**d**), which shows the lateral displacement of the needle along the *y* axis (expected uncertainty $\pm 25$ µm, one pixel), plotted with the projected area of cavitation bubbles as a function of time. Note that plots (**b,d**) share the same time axis and the numbered dashed lines in (**c**) refer to the indices of the images in (**a**).

Figure 4 illustrates a 14 gauge side cut model device, its dynamics and the cavitation activity produced by its operation in deionized water. Key moments of the spring-loaded CNB device induced cavitation activity are presented in (a) as images selected from the recorded video. The temporal correspondence of the images with the displacement of both needle and cannula tips, as well as the amount of projected cavitation, is depicted by numbered dashed lines in (c), which itself is a zoomed in time interval of interest from (d). The acoustic emission of the event is presented as a spectrogram in (b), which is also co-registered with (d). In (c) and (d) the blue and green line plots depict the y-axis (horizontal) displacement of the stylet and cannula tip, respectively. The dashed orange line with the shaded area under the curve represents the momentary orthogonal projection of the cavitation bubbles.

The strongest cavitation phenomena seem to take place around the tips of the stylet and the outer cannula, in their wake, visible at key moment 3 in Fig. 4a. It would seem that these sharp points in the needle’s geometry along the strong accelerations experienced by the needle create a suitable nucleation site for cavitation bubbles to emerge. Above the bevel, where the needle structure is cylindrical (Reynolds number *Re* in the range of 14,000–20,000), little to no cavitation is observed in the wake of the needle.

The emergence of most cavitation bubbles temporally correlates to the largest changes in the lateral displacement of the needle, i.e., when the lateral velocity of the stylet tip reaches > 7 m/s and the velocity of the cannula tip reaches > 6.5 m/s. This suggests that most of the bubbles observed in the wake of the needle structure are originating from hydrodynamic cavitation, in a similar way as it is caused by shear flows in the wake of a bluff body^14^. Around the beveled part of the inner stylet (see Fig. 2b for reference), where the needle shape presents sharp edges, the flow seems to detach more easily from the needle surface, causing the pressure to drop (decrease in the *Ca* number) and visible cavitation to emerge downstream of the needle. Moreover, when the needle is moving laterally from left to right (positive velocity along the *y* axis), the nucleation site of bubbles follows a parabolic signature along the beveled cut, visible at the key moment 4 in Fig. 4a. This spatial distribution of bubbles might transcribe the distance at which a phase transition to turbulent regime would occur, as observed by Asano et al.^13^ in the case of a cylindrical obstacle placed in a low temperature flow. Cavitation occurs even further from the needle-water interface as the width of the needle cut increases, reaching a maximum distance of 0.65 mm from the beveled cut. As the needle starts to decelerate ($t>$ 0.026 s), the amount of observable cavitation bubbles decreases. The bubbles exist periodically, lasting for milliseconds, before completely vanishing.

The spectrogram of the analysed audio signal in Fig. 4b shows bursts of broadband noise along with certain ringing frequencies. The first two bursts of broadband noise (at $\sim$ 0.015 s and $\sim$ 0.02 s) can be explained by the multi-step operation of the needle and its two different parts, the inner stylet and the outer cannula, which move separately and may collide with each other. The third burst (at $\sim$ 0.025 s) correlates to the final abrupt stop of the outer cannula and the emergence of cavitation observed around the needle tip. This burst, which is loudest of the three, reaches a peak sound pressure level of 155 dB (reference of 1 µPa) emitted at around 10 kHz. Given that all cavitation activity visually vanishes quickly after 0.027 s, the resonant frequencies, around $10-30$ kHz, persisting after this loud broadband spike could be identified as the eigenfrequencies of the needle structure evoked by the operation of the needle. Similarly induced acoustic emission was observed by Shin et al.^23^, in the case of an hydrofoil vibrated by contacting cavitation bubbles.

**Fig. 5:**
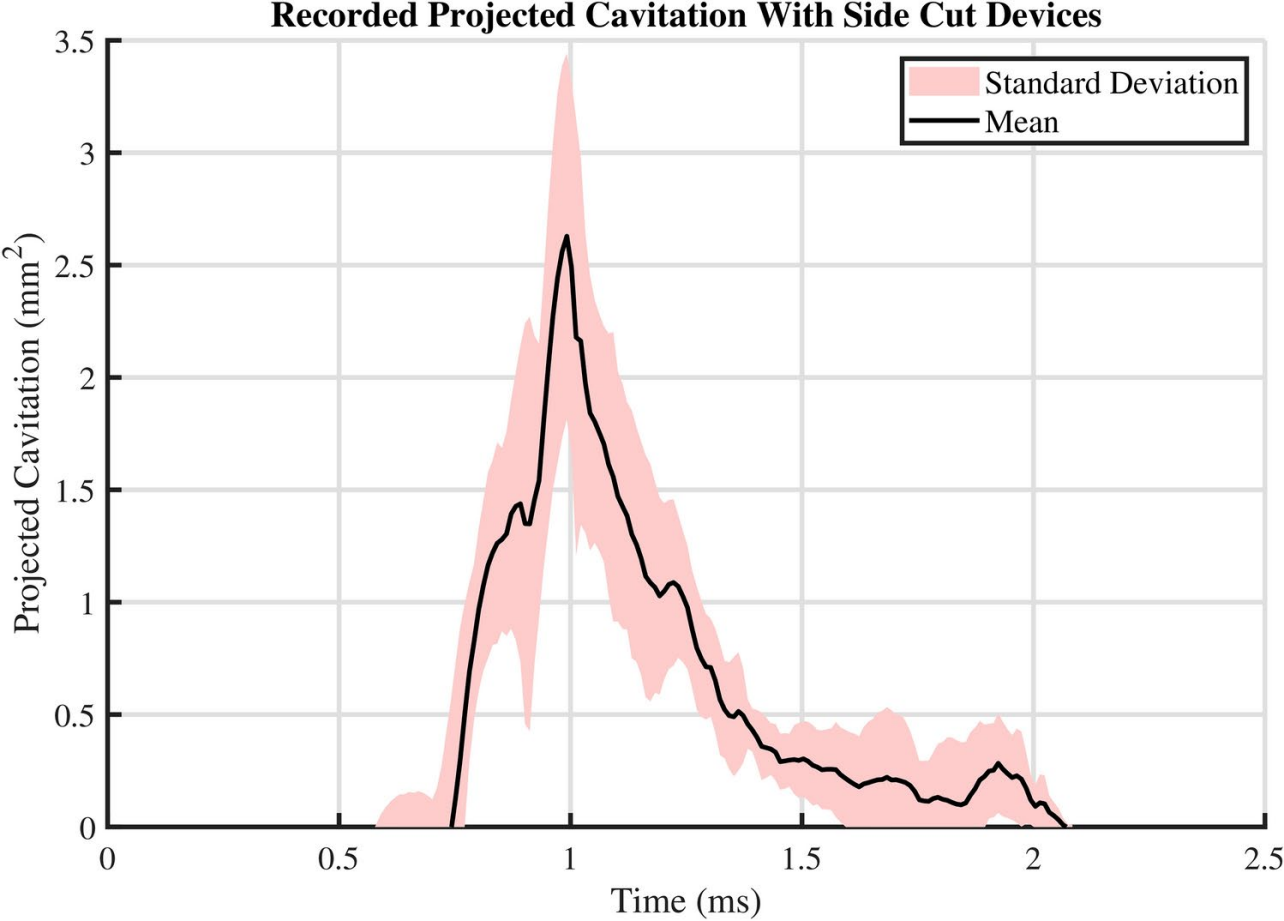
Recorded area of projected cavitation bubbles with 14 gauge side cut model needles shot inside deionized water. The dataset contains a total of $\text {N}=9$ samples from $\text {n}=3$ different devices of the same model, with 3 technical replicates for each device. The presented mean and standard deviation are calculated from all of the 9 recorded samples for each time point.

As a more statistical approach, Fig. 5 presents results showing the intensity of the recorded cavitation activity from a set of experiments with 3 different side cut devices of the same model. The mean curve computed from this dataset is comparable with the result presented in Fig. 4, demonstrating the reproducibility of the phenomenon in deionized water. The shaded area around the mean curve, depicting the standard deviation of the results in Fig. 5 indicates that the results are the most varied during the strongest cavitation activity ($\sim 0.7$ to 1.3 ms), which is to be expected, given the chaotic nature of cavitation as a phenomenon.

### Front cut needle model

**Fig. 6:**
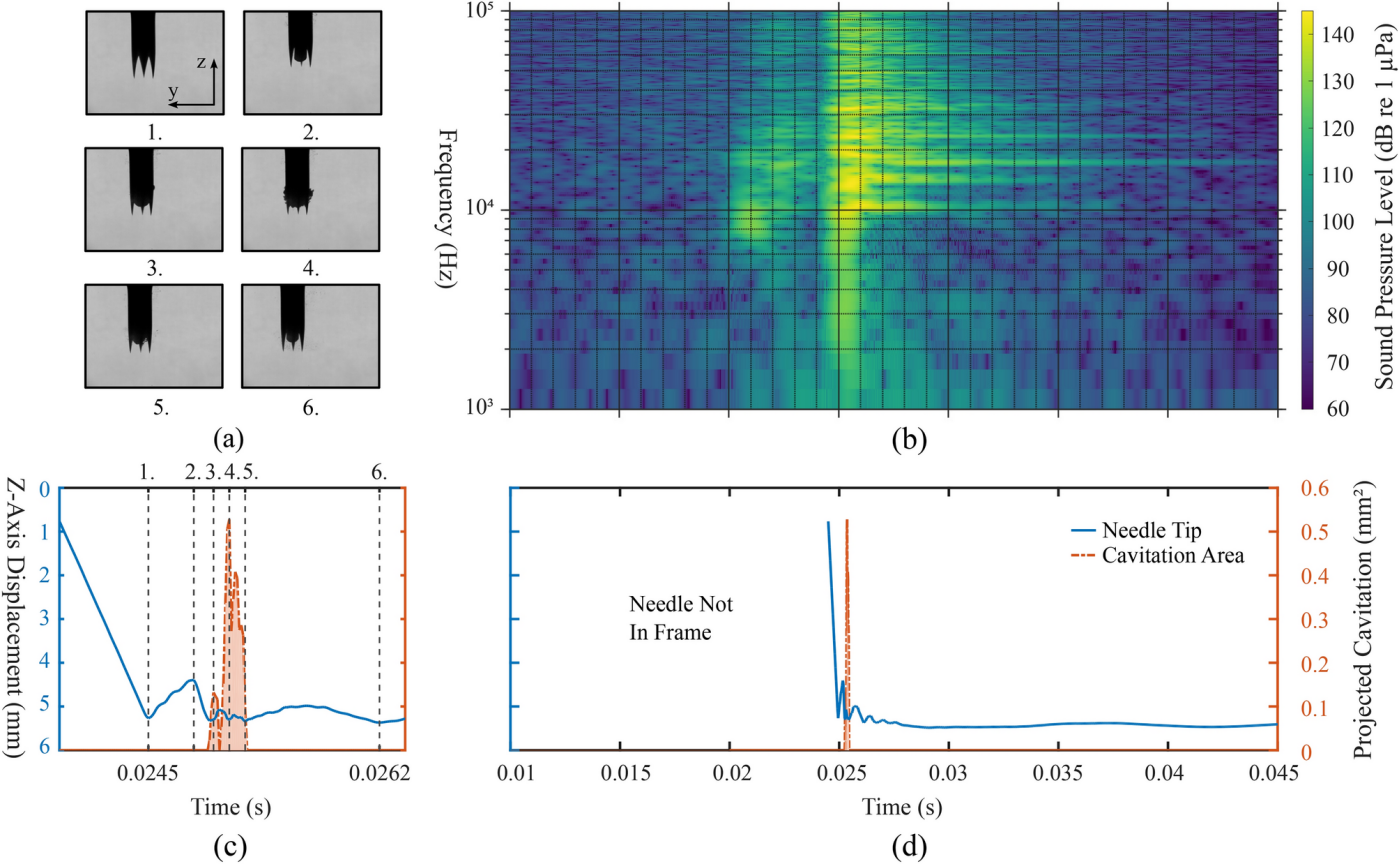
Results of a 14 gauge front cut model needle shot inside deionized water. (**a**) Images from the high speed video (Supplementary Video 2, total frame size 9.6 mm $\times$ 7.2 mm). (**b**) Spectrogram of the acoustic emission. (**c**) Time interval of interest from (**d**), which shows the vertical displacement of the needle along the *z* axis (expected uncertainty $\pm 25$ µm, one pixel), plotted with the projected area of cavitation bubbles as a function of time. Note that plots (**b,d**) share the same time axis and the numbered dashed lines in (**c**) refer to the indices of the images in (**a**).

Figure 6 demonstrates a 14 gauge front cut model device, its dynamics and the cavitation activity produced by its operation in deionized water. Unlike previously, the figure presents the z-axis (vertical) displacement of the needle tip, given that the front cut model needle does not experience any major lateral motion during regular operation ($s<$ 1 mm, where *s* is the maximum lateral displacement during relevant cavitation activity). This is represented by the blue line plot in (c) and (d). Again, the orange dashed line plot with shading under the curve represents the projected area of the visible cavitation bubbles.

The nature of the cavitation phenomenon observed with the front cut models is quite different to that seen with side cut model, as it seems to be evoked by a sudden acceleration (>8 $\hbox {m/s}^2$) in the vertical motion (*t* = 0.026 s) of the cutting cannula and the deployment of the coaxial pincer, as seen in Fig. 1b. Because the front cut device does not experience relevant lateral movement ($s<$ 1 mm), the possibility of hydrodynamic cavitation in the wake of the needle can be eliminated as the main explanation, yet wake from the longitudinal motion of the pincer cannot be excluded. The overall observed cavitation phenomenon itself is less prevalent and more short-lived ($\approx 0.25$ ms) than with the side cut model ($\approx 0.84$ ms). The visible cavitation bubbles briefly exist around the cannula tips and the coaxial pincer, before disappearing. From the shadow information represented by the projected cavitation, it can be estimated that the maximum amount of cavitation generated with the front cut model ($\approx 0.5$ $\hbox {mm}^2$) is about 7 times less than with the side cut model ($\approx 3.5$ $\hbox {mm}^2$).

The audio signal recorded from the action of the front cut model device suggests similar behavior compared to that of the side cut model. The spectrogram shown in Fig. 4b presents similar prolonged presence of frequencies, which again suggests the presence of eigenfrequencies of the vibrating needle structure in the acoustic emission. Similarly, the recorded broadband noise could be explained by the moving cutting cannula followed by the final abrupt stop along the deployment of the coaxial pincer. Note that there are only two broadband spikes, at times $t=$ 0.020 s and 0.025 s, unlike with the side cut example, which has three spikes, at times $t=$ 0.015 s, 0.020 s and 0.025 s (Fig. 4). This could be explained by the front cut model needle only having one major moving part, unlike the side cut model that has two moving parts. In addition, the primary broadband spike at $t=$ 0.025 s is not as loud as the side cut example, reaching a lower 143 dB (reference of 1 µPa), versus 155 dB recorded with the side cut model. However, the highest sound pressure levels recorded from the burst are detected on a range of frequencies (10–30 kHz), differing from its counterpart in the side cut example, where the peak emission occurs in a more narrow bandwidth.

**Fig. 7:**
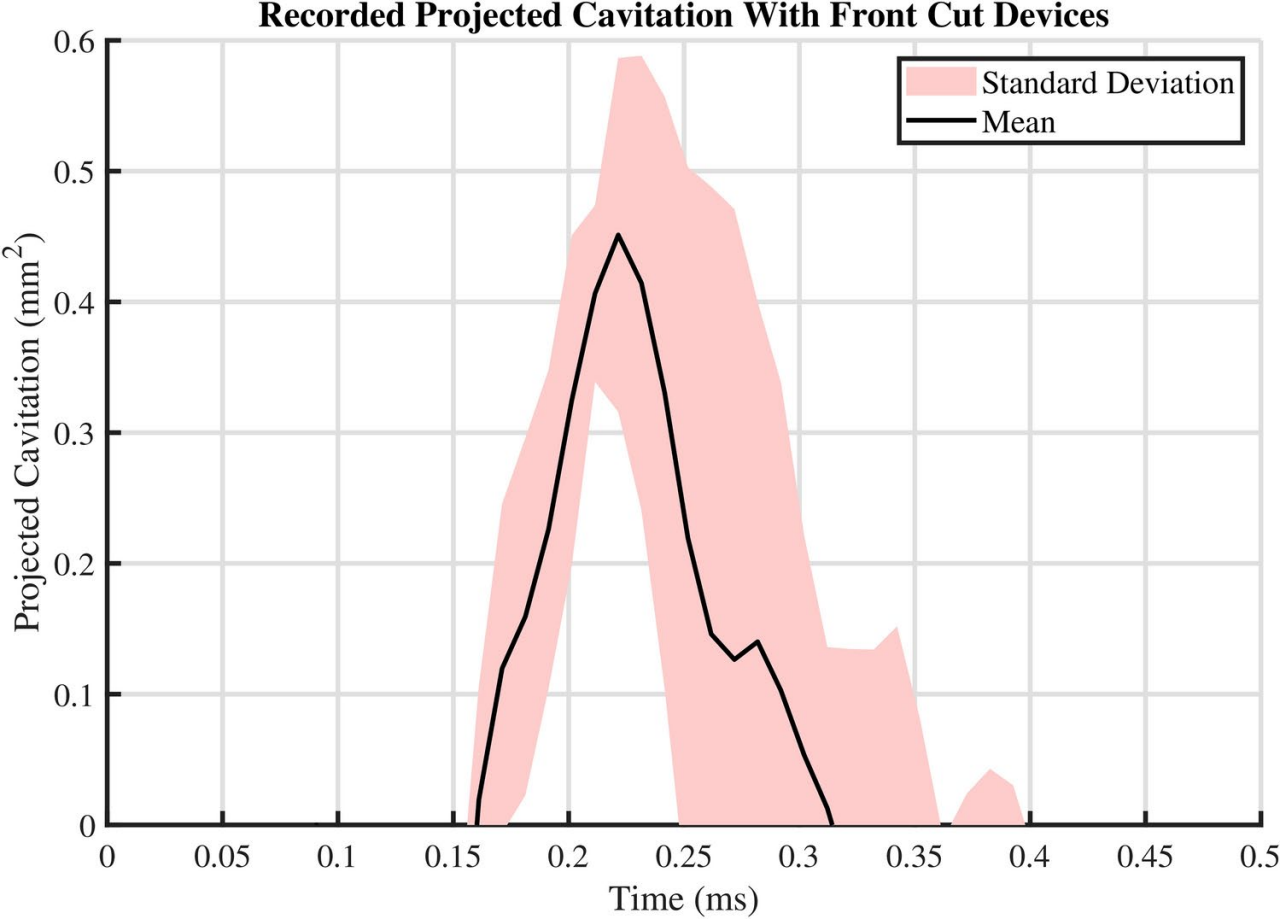
Recorded area of projected cavitation bubbles with 14 gauge front cut model needles shot inside deionized water. The dataset contains a total of $\text {N}=9$ samples from $\text {n}=3$ different devices of the same model, yielding 3 technical replicates for each device. The presented mean and standard deviation are calculated from all of the 9 recorded samples for each time point.

Similarly to the side cut results, Fig. 7 presents the projected cavitation bubble areas from a set of experiments conducted with 3 different front cut devices of the same model. The presented curve of the mean from all the samples is comparable to the curve found in Fig. 6. This demonstrates the reproducibility of the phenomenon in deionized water also with the front cut model. The standard deviation of the dataset increases considerably after the complete emergence of the first major cavitation bubble cloud ($\sim 0.22$ ms). This corresponds to the time point 4 in Fig. 6a, c.

### Cavitation in agarose gel

**Fig. 8:**
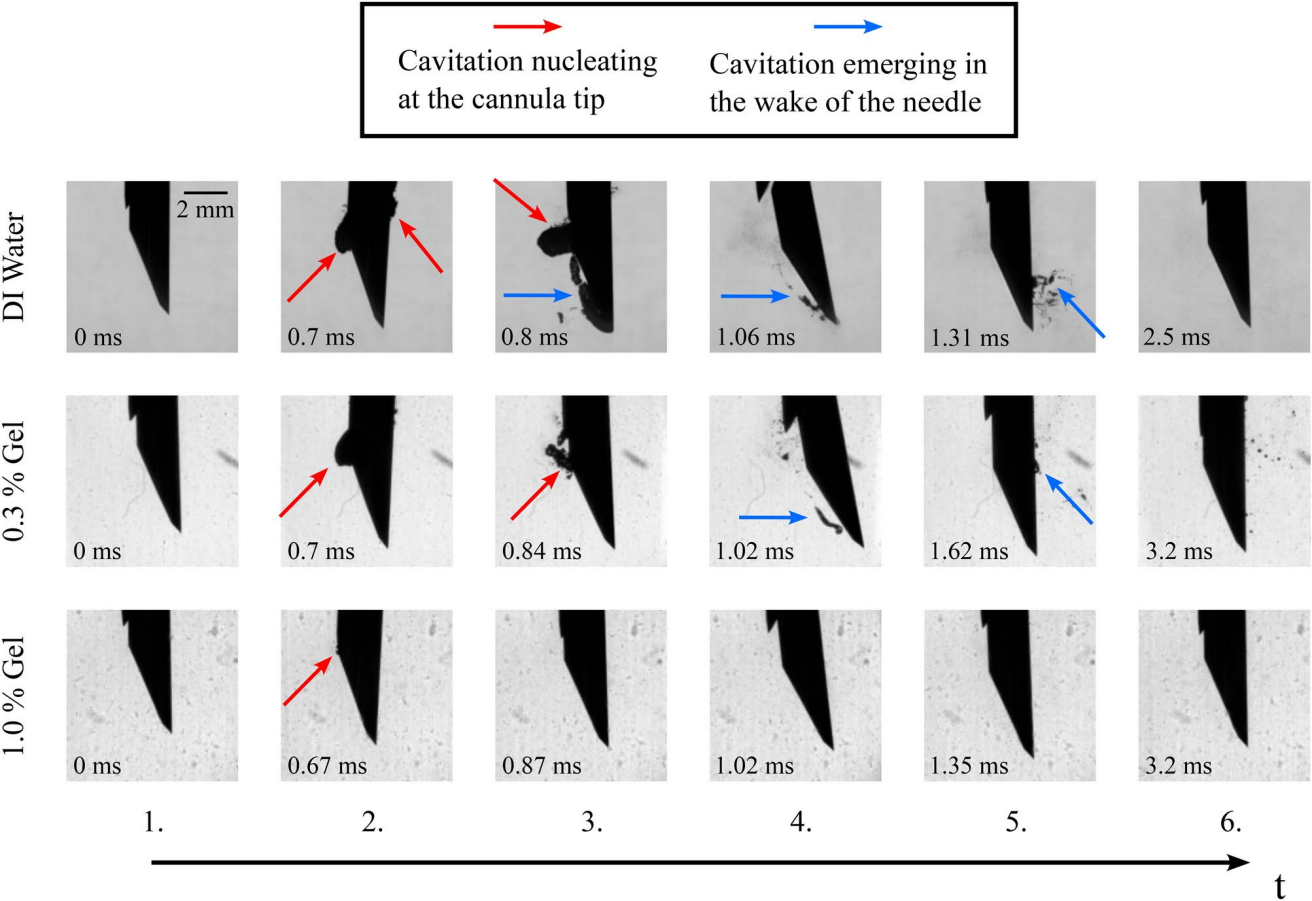
Close-up views of key image frames as a time series from the recorded high-speed video of a 14 G side cut model device tested with 0.3 % & 1.0% w/v agarose gel in comparison to the deionized water case. The results suggest that the intensity of cavitation decreases with increasing agarose concentration. The deionized water images are the same ones found in Fig. 4a.

Figure 8 compares visible cavitation activity at key time points between deionized water and both 0.3% and 1.0% w/v agarose gel with a 14 G side cut model. The key time points for each group are presented in chronological order, the exact time of the snapshots is depicted on each image respectively. The numbering of the snapshots relates to the numbering of the snapshots found in Fig. 4, as the same points of interest are chosen to represent the relevant cavitation activity.

Clear cavitation bubbles can be observed in the case of 0.3% w/v agarose gel, whose the location and spatial distribution follow a similar trend to the deionized water example, but less substantial. As the viscosity is increased (1.0% w/v), optically detectable cavitation activity becomes scarcer. An explaining factor for the different likelihoods for cavitation activity in the different concentration gels could be the contrast of compression modulus^17^, which is 1.5 kPa for the 0.3% gel and 38 kPa for the 1.0% gel^18^. Stiffer tissue is expected to resist bubble activity more than highly compliant tissues. Soft tissues, which can have a relatively low compression modulus, e.g. 2–5 kPa for lung, fat and heart tissue^17^, could be considered locations for cavitation activity with elevated risk, when subjected to a spring-loaded CNB device. Another consideration regarding the reduction of cavitation observed in the wake of the side cut needle flowing in gel concerns the density and viscosity higher than in the case of pure water. As a result, the friction at the water-needle interface would presumably increase, explaining the lower lateral velocities recorded ($u_{\text {DI water}}$ = 7–10 m/s vs $u_{\text {1.0\% gel}}$ = 4–6 m/s). This results in lower flow velocity around the needle, thus increasing the value of the cavitation number *Ca*. The Reynolds number *Re* would decrease in this situation, as in addition to the decrease in flow velocity, the kinematic viscosity ($\nu = \mu / \rho$) of agarose gel would be greater than that of water ($\nu _{\text {0.9\% gel}} = 5*10^{-6}$ $\hbox {m}^2$/s^24^ $> \nu _{\text {water}} = 10^{-6}$ $\hbox {m}^2$/s). These changes in the characterizing numbers support the experimental observation of less prevalent hydrodynamic cavitation.

**Fig. 9:**
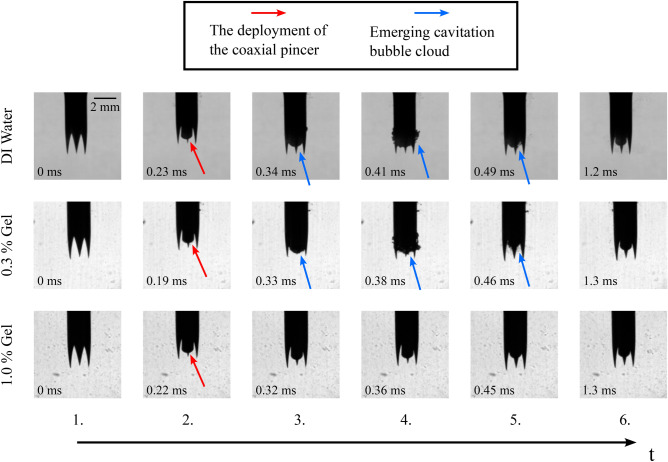
Close-up views of key image frames as a time series from the recorded high-speed video of a 14 G front cut model device tested with 0.3 % & 1.0% w/v agarose gel in comparison to the deionized water case. The results suggest that the intensity of cavitation decreases with increasing agarose concentration. The deionized water images are the same ones found in Fig. 6a.

Figure 9 compares the key moments of visually detectable cavitation activity between deionized water and both 0.3% and 1.0% w/v agarose gel using a 14 G front cut needle. Again, the snapshots of all experimental groups are in chronological order, with the exact time stamps presented on each image. The numbering of the snapshots relates to the numbering of the snapshots found in Fig. 6, as the same points of interest are chosen to represent the relevant cavitation activity.

We observe a similar trend with the front cut model to that of the side cut model, when it comes to the emergence of cavitation bubbles in the agarose phantoms. The lower concentrated 0.3 % w/v agarose gel still allows cavitation activity to emerge, again similar to the activity observed in deionized water. However, no major cavitation activity is present in the more dense 1.0 % w/v agarose gel. Overall, regarding the onset of cavitation, the results between the side cut and front cut models appear to be coherent. Both models have a likelihood to generate the cavitation phenomenon in low (0.3% w/v) concentration agarose gel, in addition to deionized water.

## Implications of cavitation observations

The ballistic properties of the operation of spring-loaded CNB devices indeed seem to have a tendency to induce cavitation in low viscosity media. If the phenomena would be found to take place in vivo, the observation could have implications to the risk assessment related to CNB procedures, especially in low compression modulus soft tissues as well as fluids such as blood or cysts, since cavitation in tissue is commonly associated with potential tissue damage.

A known adverse effect for CNB procedures is the generation of hemorrhage. A study by Chassagnon et al.^25^ found that, in transthoracic CNB operations, 58% of the patients experienced alveolar hemorrhage complications in the lung. The presence of cavitation bubbles could be a relevant risk factor especially in these transthoracic operations, due to the low compression modulus of lung tissue (2–5 kPa)^17^ and the gas-filled alveoli that could react to oscillating pressures.

The needles used in these side cut style spring-loaded CNB devices are likely to generate cavitation due to their asymmetric structural shape. The side notch displaces the center of mass from the geometrical center line of the inner stylet, promoting their lateral motion. A needle design more symmetric to the center axis could reduce the forcing of the needle to move laterally, subsequently reducing the likelihood for cavitation. Cavitation is a threshold phenomena, and we recorded lateral velocities of around 10 m/s during the cavitation events regarding the wake of the needle. After 0.025 s in Fig. 4c, the lateral velocity of both the stylet and the cannula tips remain below 5.5 m/s and no cavitation activity is observed. A threshold of lateral velocity, beyond which cavitation can occur in aqueous media, is therefore expected between 5.5 and 10 m/s. Re-engineering the needle geometry with the potential cavitation in mind could be considered in case where cavitation is undesired, while avoiding to compromise the ability of the needle to efficiently capture high quality tissue samples.

The front cut model devices employ a more symmetric needle structure in respect to the needle center axis that does not experience the strong lateral accelerations during operation like the side cut model does. This essentially eliminates most considerations for the cavitation emerging in the wake of the needle, as suggested by our results. Instead, we observe the cavitation in and around the cannula tips and the coaxial pincer, acting similarly to the cavities emerging at the cannula tip in the side cut example, both only existing for a brief period ($\approx 250$ µs) right after the sudden stop of the cannula after firing. This could be explained by the impulse generated by the mechanism stopping the needle, after it has travelled down to the tip of the needle structure inducing violent and stochastic accelerations and vibrations at the tip.

All side cut model devices considered in this study employed a lancet point inner stylet with a beveled tip outer cannula. The considered front cut models were all designed with a Franseen tip cannula. Other needle tip geometries exist and are also employed in commercial CNB devices. The design of the needle tip geometry could be a relevant factor in the observed cavitation activity and therefore a possible point of improvement in the design of CNB devices.

This work was limited to the investigation of spring-loaded CNB device induced cavitation in deionized water and tissue-mimicking matter with side cut and front cut CNB devices. As the present study focused on investigating bubble dynamics in tissue mimicking materials only, the presence and potential mechanical effects of the bubbles should be investigated in tissue, especially focusing on the effects on cellular and tissue architecture. This would be relevant as cavitation-induced tissue damage in malignant tumor could contribute to release of pathological cells and to the tumor cell seeding along the needle tract, which are known to be a concern in needle biopsy^26,27^. Importantly, the created bubbles could serve as a way to promote enhanced echogenicity in ultrasound imaging^28,29^, e.g., in image-guided CNB. This could allow to visualize the location of the sampling site and improve the traceability of sampling locations during the biopsy process.

## Conclusion

This experimental work reports on observed cavitation phenomena related to the operation of spring-loaded CNB devices. Since cavitation is well known for its potentially destructive effects in biological environments^11^, the investigation was performed not only in deionized water but also in tissue phantoms made of agarose allowing visual access to bubble dynamics. Cavitation occurred around the sharp edges of the needle more likely in less viscous environments. For the investigated needle types, the interpretations of recorded acoustic emission were twofold. Broadband noise, typically associated with inertial cavitation phenomena, can also arise from the device-internal metal-to-metal impulse-like collisions providing limited specificity to the main source of broadband sound emission. The long lasting ringing at selected frequencies around 10-20 kHz are most likely associated with the needle resonances rather than bubble resonances; the ringing of bubbles can mostly be excluded as these frequency spikes are still present long after all bubbles have visually vanished.

The clinically more broadly used side cut needle type has a greater likelihood to generate cavitation due to its asymmetric structure causing it to deflect and oscillate flexurally. However, CNB devices are widely used globally, so even if cavitation would be present in clinical use of CNB, the safety profile is still considered broadly acceptable. There is still the need to improve the safety of these devices, since complications such as hemorrhage and cancer cell seeding are not unusual^1,25^. Cavitation would be most likely to occur in low viscosity environments such as blood or cystic lesions than in high viscosity targets. Potential applications of cavitation-inducing CNB needles could include visualization of the sampling location providing traceability for sampling locations during a single session including several biopsy passes.

In conclusion, cavitation was observed in low-viscosity media such as water and agarose-based tissue phantoms, especially with side cut needles. Existence of cavitation in clinical use of CNB devices cannot be excluded. Therefore, cavitation as a physical phenomenon should be included into risk assessments in the development of CNB devices.

## Supplementary Information

Supplementary Legends.

Supplementary Video 1.

Supplementary Video 2.

## Data Availability

Curated datasets are available for download at https://zenodo.org/records/15180357. The detailed information on the used devices and methods can be requested from the corresponding author.
