# Supplementary material for: Cavitation activity induced by spring-loaded core needle biopsy devices
Source: Sci Rep. 2025 May 6;15:15825. doi: 10.1038/s41598-025-97497-z (PMC12055970; doi:10.1038/s41598-025-97497-z)
Supplement: Supplementary file 1 — Supplementary Legends. [file 41598_2025_97497_MOESM1_ESM.pdf]

Information on the supplementary materials of the manuscript “Cavitation activity induced by spring-loaded core needle biopsy devices”

Supplementary Video 1: Operation of a 14G side cut core needle biopsy device in deionized water

The video file shows exemplary action of a 14G side cut core needle biopsy device recorded in deionized water with high-speed camera shadowgraphy. Cavitation activity is recorded in the vicinity of the needle tip after the complete deployment of the outer cutting cannula of the device.

Supplementary Video 2: Operation of a 14G front cut core needle biopsy device in deionized water

The video file shows exemplary action of a 14G front cut core needle biopsy device recorded in deionized water with high-speed camera shadowgraphy. Cavitation activity is recorded in the vicinity of the needle tip after the complete deployment of the coaxial pincer of the device.
