# Supplementary figures and images for: Cavitation activity induced by spring-loaded core needle biopsy devices
Source: Sci Rep. 2025 May 6;15:15825. doi: 10.1038/s41598-025-97497-z (PMC12055970; doi:10.1038/s41598-025-97497-z)

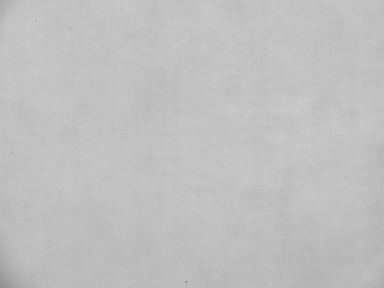

Supplement: Supplementary file 2 — Supplementary Video 1. [file 41598_2025_97497_MOESM2_ESM.gif]

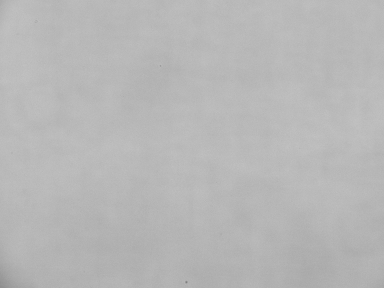

Supplement: Supplementary file 3 — Supplementary Video 2. [file 41598_2025_97497_MOESM3_ESM.gif]
